# Socio-economic and demographic determinants of under-five mortality in rural northern Ghana

**DOI:** 10.1186/1472-698X-14-24

**Published:** 2014-08-21

**Authors:** Edmund Wedam Kanmiki, Ayaga A Bawah, Isaiah Agorinya, Fabian S Achana, John Koku Awoonor-williams, Abraham R Oduro, James F Phillips, James Akazili

**Affiliations:** 1 Navrongo Health Research Centre, Ghana Health Service, Upper East Region, Box 114, Navrongo, Ghana; 2 Department of Population and Family Health, Mailman School of Public Health, Columbia University, Columbia, USA; 3 Upper East Regional Health Directorate, Ghana Health Service, Bolgatanga, Ghana

**Keywords:** Under-five mortality, Socio-economic, Demographic, Determinants, Rural, Ghana

## Abstract

**Background:**

In spite of global decline in under-five mortality, the goal of achieving MDG 4 still remains largely unattained in low and middle income countries as the year 2015 closes-in. To accelerate the pace of mortality decline, proven interventions with high impact need to be implemented to help achieve the goal of drastically reducing childhood mortality. This paper explores the association between socio-economic and demographic factors and under-five mortality in an impoverished region in rural northern Ghana.

**Methods:**

We used survey data on 3975 women aged 15–49 who have ever given birth. First, chi-square test was used to test the association of social, economic and demographic characteristics of mothers with the experience of under-five death. Subsequently, we ran a logistic regression model to estimate the relative association of factors that influence childhood mortality after excluding variables that were not significant at the bivariate level.

**Results:**

Factors that significantly predict under-five mortality included mothers’ educational level, presence of co-wives, age and marital status. Mothers who have achieved primary or junior high school education were 45% less likely to experience under-five death than mothers with no formal education at all (OR = 0.55, p < 0.001). Monogamous women were 22% less likely to experience under-five deaths than mothers in polygamous marriages (OR = 0.78, p = 0.01). Similarly, mothers who were between the ages of 35 and 49 were about eleven times more likely to experience under-five deaths than those below the age of 20 years (OR = 11.44, p < 0.001). Also, women who were married had a 27% less likelihood (OR = 0.73, p = 0.01) of experiencing an under-five death than those who were single, divorced or widowed.

**Conclusion:**

Taken independently, maternal education, age, marital status and presence of co-wives are associated with childhood mortality. The relationship of these indicators with women’s autonomy, health seeking behavior, and other factors that affect child survival merit further investigation so that interventions could be designed to foster reductions in child mortality by considering the needs and welfare of women including the need for female education, autonomy and socioeconomic well-being.

## Background

Under-five mortality decline has improved from 1.2% per year between 1990 and 1995, to 3.9% per year between 2005 and 2012 [1]. In spite of this substantial drop in global child mortality rate, about 6.6 million children still die every year before their fifth birthday worldwide which implies 18,000 under-five children die each day [1]. There are huge disparities in child mortality among low and middle income countries and the industrial world with Sub-Saharan Africa and South East Asia carrying the highest burden of under-five mortality [2-4].

Children in sub-Saharan Africa have the highest risk of death in the first month of life and are still leading in under-five mortality rates with one in every nine children dying before their fifth birthday as of 2011 [2].

It is worth noting that the year 2008 recorded a rate of one in seven children (144 per 1000 live births) dying before their fifth birthday with the highest levels occurring in West and Central Africa. Within 34 countries where under-five mortality exceeded 100 out of 1000 live births in 2008, all except one are in sub-Saharan Africa [5]. The rate of improvement in child survival in Sub-Saharan Africa is insufficient to meeting United Nations Millennium Development Goal 4 of reducing under-five mortality rate by two-thirds between 1990 and 2015 as it has the highest risk of death in the first month of life and is among the regions showing the least progress globally [1,2].

In order to accelerate the decline in under-five mortality rate, specific proven interventions would have to target important causes of child death [6-8]. Since no single factor can account for the high child mortality [9], in developing these interventions there is the need to understand the multiplicity of factors that determine child mortality especially in resource poor settings [10,11].

Previous studies have shown that various factors influence child health and survival including place of residence, mothers age, mother’s education, place of delivery, birth order, sex of child, religion of parents, household headship and household socio economic status [9,12-18].

Though poverty is well acclaimed as an essential factor influencing child mortality [4,14,19,20], findings on the effect of household socio economic differentials on child mortality have been mixed. A study in parts of rural Ghana and another in Tanzania did not find any significant effect of household socio economic status on child mortality [18,19] while a study using Nigeria Demographic and Health Survey for 2008, found that relatively prosperous households were less likely to experience child death than the poorest households in rural Nigeria [13].

Existing literature has documented mixed results, such as place of delivery, birth order and sex of child. For instance, abundance of evidence suggest that women who deliver at health facilities have a lower chance of child death as compared to those who deliver at home due to the use of skilled delivery at health facilities and the none existence of such at home [6,12-14]. However, studies in South Africa and parts of Nigeria suggest that place of delivery does not have significant effect on either perinatal or under-five mortality [16,17].

In Ghana, just as in many other countries in Sub-Saharan Africa with low socio economic development, under-five mortality is relatively high with a recent reported national figure of 90 per 1000 live births [21]. Moreover within Ghana, disparities exist in under-five mortality rates between regions. In the more resource rich regions of southern Ghana, under-five mortality rate ranges from 75 per 1000 live births whiles in the most impoverish and deprived regions of the north such as the Upper East Region it is as high as 128 per 1000 live births [21]. Therefore there is the need for concerted efforts especially in resource poor settings if we hope to achieve the desired improvements in under-five mortality.

Due to disagreements in existing literature on the influence of particular social, economic and demographic attributes of mothers on under-five mortality coupled with sparse information on the topic in the Ghanaian setting, this paper further examine social, economic and demographic factors that influence under-five deaths and the magnitude of their influence in a rural impoverished setting in northern Ghana. It is hoped that findings from this paper will contribute to literature on under-five mortality in rural poor settings and perhaps contribute to identifying priority interventions to address the persistent mortality in settings such as this.

## Methods

### Study setting

The study was carried out in the Upper East Region of Ghana. Located in the northernmost part of Ghana, the region has a population of about 1,046,545 with an annual growth rate of 1.2% [21]. The region is predominantly rural, with 79% of its residents located in rural areas and as high as 72.3% of households are headed by males, total fertility rate is 3.43 and under-five mortality stands at 128 per 1000 live births [21]. The region is made up of about seven ethnic groups all of which are patriarchal in nature. Social research has documented customs of gender stratification that constrain female autonomy with respect to decision making, household governance and health seeking behavior [22]. Subsistence agriculture constitutes the mainstay of the economy of this region contributing as high as 83.7% of households’ economic activity [21,22]. The area is characterized by two main seasons, a short rainy season from May to September and a long dry season from October to April. With such pervasive dependence on subsistence rain-fed agriculture, the region ranks among Ghana’s most impoverished regions [19,22,23], with low literacy and a per capita income level that is estimated to be about a quarter of the level estimated for Ghana as a whole [24].

### Study design

This paper makes use of data from a cross-sectional baseline survey of the Ghana Essential Health Intervention Project (GEHIP) conducted in 2011. The purpose of this survey was to provide the project team with detailed information on maternal and child health, fertility, family planning, universal health coverage and other basic health indicators for eventual evaluation of the project.

GEHIP is a five year health systems strengthening and research program being conducted in the Upper East region of Ghana. It seeks to improve Ghana’s comprehensive primary healthcare initiative, known as the Community-based Health Planning and Services Program (CHPS). CHPS provides a wide range of essential preventive and curative services to some of Ghana’s most rural and impoverished locations but has however been faced with serious service gaps and operational flaws preventing it from achieving its full potential [20]. The GEHIP strategy includes introducing a series of training and technical assistance programs aimed at strengthening the capacity of the health systems. Also, additional maternal and child health interventions are being added and increased support provided to system structures to enhance overall effectiveness [20]. GEHIP is being implemented by the Upper East Regional Health Directorate of the Ghana Health Service (GHS) with technical assistance from the Mailman School of Public Health, Columbia University and the school of Public Health of the University of Ghana. Navrongo Health Research Center (NHRC) has the responsibility to provide data for the evaluation of GEHIP.

With the exception of the Kassena-Nankana East and West Districts which are research sites of the Navrongo Health Research Centre (NHRC), the other seven districts in the region as of 2011 were involved in the survey. The districts include: Bawku East Municipality, Bawku West, Talensi-Nabdam, Garu-Tempane, Bongo, Builsa and Bolgatanga Municipality. The sampling unit of the survey was the household, which is defined as ‘a person or group of persons living together in the same house or compound, sharing the same housekeeping arrangements and being catered for as one unit’ [25]. The sample frame was obtained from the Ghana Statistical Service (GSS). A two-stage sampling procedure was involved, the GSS sampled 66 enumeration areas (EAs) and Probability sampling proportional to population size was used, all women age 15–49 years in sampled households were eligible to be interviewed using a structured questionnaire. A total of about 5400 women were interviewed, however, this paper is based on 3,975 of the women representing those who have ever given birth.

### Socio-economic and demographic variables

The variables used in this study included mother’s age, mothers’ highest level of education, rural/urban residence, mother’s marital status, occupation, religion, autonomy, mother’s involvement in polygamous marriage, contraceptive use, national health insurance registration status and household socio economic status.

### Data analysis

STATA 11.2 was used for all the analysis. For the purpose of this paper, the unit of analysis is respondents who had experienced child birth and the measurable outcome was the experience of under-five death. Mothers’ age was recoded into three categories (15–19, 20–34 and 35–49). Mother’s highest level of education was also categories into three: those whose highest level of education was primary or junior high school were merged into one category while senior high school and tertiary were also merged into another. Respondents’ marital status was recoded: all who had reported to be single, widowed, divorced or separated were merged and labeled as ‘not married’. Place of residence was also recorded as either urban or rural. Occupation was recoded into five categories: all respondents who were involved in trading, craftsmanship, dressmaking etc. were merged and classified as “self-employed”. The other categories under occupation are: farming, government employed, student and others.

In determining the variable autonomy, a question was posed to respondents as to who often makes decisions about major household purchases and this was recorded, respondents who answered that they personally made those decisions themselves and those who answered that they do that jointly with their husbands were merged together and considered to be autonomous while respondents who said that it was the sole prerogative of their husbands and those who answered that someone else other than they or their husbands, were merged and considered ‘not autonomous’.

We used principal component analysis (PCA) a multivariate approach to compute wealth index by using household assets as a proxy to estimate household socioeconomic status. In all 13 household items (assets) were involved in the analysis and wealth index was classified into 3 socio-economic tertiles namely: relatively poor, middle and rich.

Bivariate analysis using chi square test was used to test for association between the independent outcome variables and ever experiencing under-five death. All variables that showed significant association (p < 0.05) in the bivariate analysis were then included in the multivariate analysis (logistic regression model). We calculated odds ratios with each covariate. Variables used in the logistic regression were first tested for multi co-linearity using the variance inflation factor (VIF) and this was found not to be a problem with an average VIF of 2.54 (VIF more than 20 indicates multi co-linearity). In calculating the odds ratio for each category of the independent variables, the first group was always taken as the reference category.

### Ethical consideration

Before the baseline was conducted for GEHIP, ethical clearance was sought from ethics committees of the Ghana Health Service and the Navrongo Health Research Centre Institutional Review Board (NHRC IRB). Inform Consent was obtained from each participant. Also data sets used were anonymous of participant identity.

## Results

### Characteristics of study participants

In all 3,975 respondents had ever experienced child birth. Of this number 1,481 representing 37.3% had ever experienced under-five child death. 3.7% were between 15–19 years, about half (50.9%) were between 20–34 years whiles 45.4% were between 35–49 years.

Only 3.1% of respondents had attained education up to senior high school or above while 75.6% had no formal education at all. About 12.9% where either not married, widowed, divorced or separated while 87.1% of respondents were observed to either be married or live with a man as if married. As high as 87.5% were observed to be rural dwellers, while 12.5% were resident in urban settings. With regards to religious affiliation, 51.8% belong to Christianity while 27.1% and 16.9% belong to Islamic and traditional religions respectively. In terms of occupation, 46.3% of respondents were engaged in farming, 36.5% were self-employed while only 12.1% were civil servants and less than 2% were students.

As high as 64.1% could be described as being “autonomous” that is: they make their own decisions about major household purchases or jointly with their husbands while 35.9% of respondents rely solely on their husbands or another relative to make such decisions. Also 34.1% of respondents were found to be in polygamous unions; that is their husbands have more than one partner. 58.8% of respondents were registered under the Ghana national health insurance scheme.

According to our wealth index on socio economic status using common household assets in rural northern Ghana, 43.8% were relatively poor, 20.8% were within the middle class while 35.4% were within the rich tertile. In terms of contraceptive use, 15.2% of respondents reported to be using contraceptives to delay or avoid getting pregnant. Table 1 below gives a detailed description of the background characteristics of the 3,975 respondents involved in this study.

**Table 1:** Background characteristics of respondents, 2011 [N = 3975]

**Background characteristics**		**Number (N)**	**Percent (%)**
***Age group***	15-19	145	3.7
	20-34	2024	50.9
	35-49	1806	45.4
***Highest Level of education***			
	None	3005	75.6
	Primary/Junior High School	846	21.3
	Secondary/Tertiary	124	3.1
***Marital status***			
	Not married (single/widowed/divorced)	511	12.9
	Married	3464	87.1
***Occupation***			
	Farming	1842	46.3
	Self Employed	1451	36.5
	Government Employed	482	12.1
	Student	68	1.7
	Other	130	3.3
	No response	2	0.1
***Religion***			
	Christianity	2057	51.8
	Traditional	670	16.9
	Islam	1078	27.1
	No response	170	4.3
***Place of residence***			
	Urban	497	12.5
	Rural	3478	87.5
***District of residence***			
	Bawku East	705	17.7
	Bawku West	432	10.9
	Bolga M.	352	8.9
	Bongo	488	12.3
	Builsa	692	17.4
	Garu/Tempani	810	20.4
	Talensi/Nabdam	496	12.5
***Financial autonomy***			
	Autonomous	2547	64.1
	Not autonomous	1428	35.9
***Polygamous partner***			
	Yes	1355	34.1
	No	2139	53.8
	No response	481	12.1
***NHIS Coverage***			
	Not registered	2338	58.8
	Registered	1637	41.2
***Socio economic status (Wealth Index)***			
	Poor	1742	43.8
	Middle	828	20.8
	Rich	1405	35.4
***Contraceptive use***			
	Yes	605	15.2
	No	2459	61.9
	No response	911	22.9

### Bivariate analyses

The results of the bivariate analysis of ever experiencing an under- five death and predictor variables are shown in Table 2 below. Respondents aged 35 years and above were more likely to experience under-five death compared to younger respondents (p < 0.001, 95% CI). Similarly, deaths among under-five children differed significantly with the level of respondents’ education, with those of relatively higher education having a lower chance of experiencing under-five death (p < 0.001). Marital status was also slightly associated with under-five death in bivariate analysis with respondents not married having a high chance of experiencing under-five death compared to those married (p = 0.04, 95% CI).

**Table 2:** Bivariate analysis of mother’s experience of child death [N = 3975]

**Determinants**	**Ever experiencing an under-five death (n = 3975)**		
	**YES**	**No**	**p-value**
	**n (%)**	**n (%)**	
**Age group**			
15-19	10(6.90)	135(93.10)	
20-34	453(22.38)	1571(77.62)	<0.001
35-49	1018(56.37)	788(43.63)	
**Highest level of education**			
None	1282(42.66)	1723(57.34)	
Primary/Junior High School	185(21.87)	661(78.13)	<0.001
Secondary/Tertiary	14(11.29)	110(88.71)	
**Marital status**			
Not married	211(41.29)	300(58.71)	0.043
Married	1270(36.66)	2194(63.34)	
**Occupation**			<0.001
Farming	753(40.88)	1089(59.12)	
Self Employed	520(35.86)	930(64.14)	
Government employed	158(32.85)	323(67.15)	
Student	5(7.35)	63(92.65)	
Other	45(34.62)	85(65.38)	
**Religion**			
Christianity	702(34.13)	1355(65.87)	
Traditional religion	302(45.07)	368(54.93)	<0.001
Islam	402(37.29)	676(62.71)	
No response	75(44.12)	95(55.88)	
**Place of residence**			
Urban	162(32.60)	335(67.40)	0.022
Rural	1319(37.92)	2159(62.08)	
**Financial autonomy**			
Autonomous	1020(40.05)	1527(59.95)	<0.001
Not autonomous	461(32.28)	967(67.72)	
**Polygamous partner**			
yes	609(44.94)	746(55.06)	<0.001
No	666(31.14)	1473(68.86)	
No response	206(42.83)	275(57.17)	
**NHIS registration**			
Not registered	951(40.68)	1387(59.32)	<0.001
Registered	530(32.38)	1107(67.62)	
**Socio economic status (Wealth index)**			
Poor	687(39.44)	1055(60.56)	
Middle	325(39.25	503(60.75)	0.001
Rich	469(33.38)	936(66.62)	
**Contraceptive use**			
Yes	215(35.54)	390(64.46)	
No	1003(40.79)	1456(59.21)	<0.001

Occupation of respondent was equally associated with experience of under-five deaths with higher proportions of those involved in farming and the self-employed experiencing under-five death compared to civil servants and students (p < 0.001, 95% CI). Religion of respondent was significantly associated with under-five death with those who practice no religion and those who practice traditional religion having higher chances of experiencing under-five death compared to those who practice Christianity and Islamic religions (p < 0.001, 95% CI). Place of residence was however slightly associated (p = 0.022, 95% CI) with rural dwellers having slightly higher proportions of under-five death experience compared to that of urban dwellers.

The association of autonomy and childhood mortality was significant with respondents regarded as autonomous rather having a higher chance of experiencing an under-five death than those considered not autonomous (p < 0.001, 95% CI).

Also, polygamous status of respondents, contraceptive use, national health insurance coverage and socio economic status of respondent’s household were all significant in the bivariate analysis (p < 0.001, 95% CI), those involved in polygamous unions had higher chance of experiencing under-five death than their counterparts, contraceptive users had a lower chance of experiencing under-five death compared to non-users while those covered under the national health insurance scheme were less likely to experience under-five death. The proportion of respondents in the rich tertile that experienced under-five death was slightly less than that of the middle class and the relatively poor tertiles.

### Multivariate analysis

Table 3 below shows the results of the multivariate analysis (logistic regression model) of determinates of under-five mortality in rural northern Ghana.

**Table 3:** Respondents experience of under-five death

**Determinants**	**Odds ratio**	** P > z**	**95% conf. interval**	
**Age group (Compared to 15–19)**				
20-34	3.00	<0.001	1.41	6.37
35-49	11.44	<0.001	5.38	24.34
**Level of education (Compared to No education)**				
Primary/Junior High School	0.55	<0.001	0.44	0.70
Secondary/Tertiary	0.24	<0.001	0.10	0.57
**Marital status (Compared with Not married)**				
Married	0.73	0.01	0.57	0.94
**Occupation (Compared with Farming)**				
Self Employed	0.91	0.28	0.76	1.08
Housewife	0.98	0.86	0.75	1.27
Government Employed	0.43	0.13	0.14	1.29
Student	1.02	0.94	0.63	1.64
**Religion (Compared with Christianity)**				
Traditional religion	1.20	0.10	0.96	1.50
Islamic religion	0.96	0.70	0.79	1.17
No response	1.10	0.62	0.76	1.60
**Residence (Compared with Urban)**				
Rural	0.82	0.16	0.63	1.08
**Financial Autonomy (Compared to Autonomous)**				
Not Autonomous	1.02	0.80	0.86	1.21
**Polygamous partner (Compared with those in polygamous unions)**				
Not in polygamous union	0.78	0.01	0.66	0.93
**Socio economic Status/wealth index (Compared to poor)**				
Middle	1.07	0.50	0.87	1.32
Rich	0.95	0.59	0.78	1.15
**National Health Insurance (Compared with registered)**				
Not registered	0.90	0.22	0.76	1.06
**Contraceptive use (Compared with users)**				
Not using contraceptives	1.14	0.22	0.93	1.40
No response	0.22	<0.001	0.09	0.52

Logistic regression model.

In the model, respondent’s age, highest level of education, marital status and polygamous status of partner were significant predictors of under-five mortality. Level of education had a statistically significant effect on experience of under-five death. For example women who had up to primary or junior high school education where 45% less likely (odds ratio = 0.55) to experience an under-five death than those who had not attained any form of formal education at all and those who had up to senior high school or tertiary education where even 76% less likely (odds ratio = 0.24) to experience under-five death than women without any formal education at all. Similarly age of respondent had a positive and statistically significant impact. Women who were between the ages of 35 and 49 years were about 11 times more likely to experience under-five death than those below the age of 20 years. On the other hand, women who were married had a 27% less likelihood (odds ratio = 0.73) of experiencing an under-five death than those who were either reported single, divorced or widowed.

Conversely, maternal occupation, religious affiliation and place of residence were not significant determinants of under-five mortality in the multivariate analysis. Autonomy, household socio economic status, enrollment in the national health insurance scheme and contraceptive use also did not confer significant odds of under-five mortality.

Being in a polygamous union however is significantly associated with under-five mortality, with children of mothers not in polygamous unions having significantly higher likelihood (22%) of survival (odds ratio = 0.78) than their counterparts.

## Discussion

In this study we have examined the influence of particular social, economic and demographic characteristics of mothers on under-five mortality in rural northern Ghana. Results showed that several factors are implicated in under-five mortality.

The bivariate analysis shows that variables such as mother’s age, level of education, marital status, place of residence, occupation, religion, autonomy, contraceptive use, national health insurance enrollment status, polygamous union and socio economic status were significantly associated with experience of under-five death in this largely rural setting. Controlling for the confounding effects of the various factors, mother’s age, level of education, marital status and polygamy, were found to have significant robust association with childhood mortality.

Both multivariate and bivariate analysis indicate that children born to older mothers (35 years and above) were more at risk of under-five death compared to younger mothers (20 years of age), this is consistent with available literature that points to the fact that maternal age is a strong predictor of child survival [12,14,18]. Reasons for the childhood mortality disadvantage for older mothers may be twofold: first, older cohort mothers are less likely to be educated and therefore, less likely to be associated with the enhanced mortality advantage associated with education, and second, older mothers are more likely to be associated with higher parity.

Level of mothers’ education emerged as a strong predictor of under-five mortality in both bivariate and multivariate analysis after controlling for other variables; this has long been acknowledged by other scholars [4,9,14,16] though a few studies suggest otherwise [12]. The effect of maternal education on child survival is over whelming and cannot be over emphasized. The reasons may be that educated mothers are more likely to receive antenatal care and are more capable of gaining access to health care. Also education can contribute to child survival by delaying marriage and motherhood hence decreasing the total number of children a woman has [12]. Warren’s findings from Ethiopia indicate that some women do not seek care due to lack of understanding of the benefits which has effects on child and maternal survival [26].

As presented in these findings, married women were less likely to experience child death than their unmarried counterparts and this was statistically significant even in the multivariate analysis. Not many studies have been done on the effect of mother’s marital status on child mortality but one can sense that mothers in stable marriages would get support from their partners during antenatal through to postnatal care which can reduce risk of child mortality as shown by our results. Also, marriage may confer advantages such as pooling of resources to either patronize good health services or provide adequate care with respect to providing good nutrition to infants and children.

Religious affiliation did not show significant odds in the multivariate analysis though the odds points to the fact that respondents who did not practice any religion and those who practice traditional religion had a higher chance of experiencing under-five death than Christians and Muslims. This is consistent with a study in parts of Nigeria which showed that children born to mothers who practice traditional religion had increased risk of about thrice than the general population to experience perinatal deaths [17].

Contrary to Ettarh and Kimani’s findings in Kenya and another study in Nigeria which showed that rural poor mothers have a higher likelihood of experiencing child death than their urban counterparts [12,17], the findings of this study did not show any significant effect of place of residence on ever experiencing under-five death. It is likely the place of residence for some respondents could have changed between the time they experienced under-five death and the time of this survey. The existence of wide spread community based health planning and services program (CHPS) in the region could also partly be the reason for which place of residence did not confer significant odds on the experience of under-five deaths.

In spite of previous studies establishing that maternal autonomy promotes health-seeking behavior for sick children with positive outcomes on child survival [9,13,27], this study did not seem to convincingly show the result of this on child survival. In this study we expected that mothers who profess to be financially autonomous would have significantly less chance of losing an under-five child but this is not so, as financially autonomous women were only 2% less likely to experience under-five death and this was not even statistically significant at the multivariate level. This may be due to our measure of autonomy. While other studies look at autonomy largely from education and employment [27] others perceive autonomy on the basis of decision making and to a large extend household headship. There is hence the need for a more concise and acceptable measure of female autonomy.

Another area which most studies have not looked at is the effect of polygamy on child mortality. This study shows that children whose mothers are in polygamous unions have 22% higher chance of experiencing under-five death than those in monogamous marriages. This may be due to possible rivalry from co-wives and neglect from husbands during pregnancy and early child care which is typical of African polygamous marriages with consequential effects on child survival. It could also be due to the fact that in this predominantly agrarian communities where women do not work and often tend to rely on the little resources that are provided by their husbands, women who are in polygamous marriages will be doubly stretched with regards to resources because the little the husband has will have to be shared among the several wives.

The effect of contraceptive use on under-five mortality though not significant at the multivariate level, it points to a 14% less likelihood of contraceptive users experiencing under-five death and a significant association in bivariate analysis. This is in line with other studies which have previously indicated a positive association of contraceptive use to low child death [9]. Therefore family planning methods are well recommended as an effective tool for birth spacing, prevention of unwanted pregnancies and affording mothers adequate time to care for the newborn and regain strength before conceiving again.

Contrary to most previous studies that had highlighted the effect of socio economic factors on child mortality [4,9,12,13,15], socio economic status did not confer significant odds in the multivariate analysis. Owais et al. in examining why families of newborns do not accept hospital care in Pakistan identified largely financial reasons as a major barrier to access and utilization of health facilities [28]. Other studies have also indicated the role of socio economic differentials in health care seeking behavior with those of higher socio economic status more likely to use health care facilities than those at lower levels [28-30]. Which would invariably have a positive impact on child survival, but this is not what we observed here. Some studies suggest that the presence of wide spread community-based health planning and services program (CHPS) in the region has the potential to reduce the effect of socio economic differential on child mortality [19,23]. Studies in Vietnam and Nigeria showed that mothers from poor settings with transportation difficulty have a higher likelihood of child death [14,17]. Therefore in spite of these findings, we recommend interventions that improve the economic wellbeing of women in this setting given the prevalence of poverty in the area.

## Conclusion

The causes of high under-five mortality in resource poor settings are complex and merit concerted efforts to clarify their implications to improve child survival. On the basis of these findings, we suggest that specific efforts with focus on under-five mortality decline need to target the individual needs and welfare of women. In order of priority, the promotion of female education, gender equity, family planning and addressing the vast socio-economic differentials in northern Ghana are important social steps to improving under-five survival.

## Competing interests

There is no competing interest.

## Authors’ contribution

EWK and JA conceived the study and participated in design and coordination of the study. EWK, JA, AB and FSA carried out the experiment. EWK, JA and IA did the data analysis. EWK, JA and AB drafted the manuscript. JFP, AO, AB, JKA critically reviewed the paper. All authors read and approved of the final manuscript.

## Pre-publication history

The pre-publication history for this paper can be accessed here:

http://www.biomedcentral.com/1472-698X/14/24/prepub
